# Health professional's perceptions of and potential barriers to smoking cessation care: a survey study at a dental school hospital in Japan

**DOI:** 10.1186/1756-0500-3-329

**Published:** 2010-12-07

**Authors:** Atsushi Saito, Makiko Nishina, Keiko Murai, Akiko Mizuno, Fumie Ueshima, Takemi Makiishi, Tatsuya Ichinohe

**Affiliations:** 1 Department of Clinical Oral Health Science, Tokyo Dental College, 2-9-18 Misaki-cho, Chiyoda-ku, Tokyo, 101-0061, Japan; 2 Department of Internal Medicine, Tokyo Dental College, 2-9-18 Misaki-cho, Chiyoda-ku, Tokyo, 101-0061, Japan; 3 Section of Nursing, Tokyo Dental College Suidobashi Hospital, 2-9-18 Misaki-cho, Chiyoda-ku, Tokyo, 101-0061, Japan; 4 Section of Dental Hygiene, Tokyo Dental College Suidobashi Hospital, 2-9-18 Misaki-cho, Chiyoda-ku, Tokyo, 101-0061, Japan; 5 Department of Dental Anesthesiology, Tokyo Dental College, 1-2-2 Masago, Mihama-ku, Chiba, 261-8502, Japan

## Abstract

**Background:**

Smoking is currently accepted as a well-established risk factor for many oral diseases such as oral cancer and periodontal disease. Provision of smoking cessation care to patients with oral problems is a responsibility of health care professionals, particularly dentists and dental hygienists. This study examined the smoking-related perceptions and practices of dental school hospital-based health professionals in Japan.

**Findings:**

A cross-sectional study design was used. The sample was formed from dentists, dental hygienists, physicians and nurses of a dental school hospital in Tokyo, Japan (n = 93, 72%). Participants were asked to complete an 11-item questionnaire assessing demographic variables and smoking history, provision of smoking cessation advice or care, attitudes about smoking cessation, and perceived barrier(s) to smoking cessation care. Eighteen percent of participants reported being current smokers and 15% reported being ex-smokers, with higher smoking rates reported by dentists compared with other health professionals (p = 0.0199). While recognizing the importance of asking patients about their smoking status, actual provision of smoking cessation advice or care by participants was relatively insufficient. Interventions such as 'assess willingness to make a quit attempt' and 'assist in quit attempt' were implemented for less than one-quarter of their patients who smoke. Non-smokers were more likely to acknowledge the need for increased provision in smoking cessation care by oral health professionals. 'Lack of knowledge and training' was identified as a central barrier to smoking cessation care, followed by 'few patients willing to quit'.

**Conclusions:**

A need for further promotion of smoking cessation activities by the health professionals was identified. The findings also suggest that dentists and dental hygienists, while perceiving a role in smoking care, do require training in the provision of smoking cessation care to hospital patients. In order to overcome the potential barriers, it is necessary to provide staff with appropriate training and create an atmosphere supportive of smoking cessation activities.

## Background

Tobacco use is one of the leading causes of preventable mortality in industrialized nations [1]. In 2002, World Health Organization [2] reported that about one in eight deaths in Japan are due to smoking (about 100,000 deaths a year), and Japan had some of the most lenient anti-tobacco laws among developed nations. Since then, considerable efforts have been made to promote smoking cessation activities. The national project, 'Health Japan 21' includes tobacco control, and the prevention of passive smoking was included in the Health Promotion Law implemented in 2003 [3]. Furthermore, in 2006, smoking cessation treatment by physicians was approved under the national health care insurance in Japan *via *the recognition of 'nicotine dependence' as a disease [4]. However, in 2008, it was shown that 36.8% of Japanese adult men and 9.1% of women still smoke regularly [5]. Given this situations, it is important for health professionals in Japan to further promote smoking cessation program.

Smoking is accepted as a well-established risk factor for many oral diseases including oral cancer and periodontal disease [6]. Oral health professionals are in a unique position to advise smokers to quit by providing effective counseling on the various aspects of tobacco-induced diseases. Dentists and dental hygienists have regular contact with smokers and a great potential for helping their patients to quit smoking; yet, this potential is often underutilized [7-14]. It has also been suggested that Japanese health professionals may lack sufficient awareness of their position as role models for dental patients, in comparison to western developed countries [15,16].

Concurrently with the inclusion of smoking cessation intervention by physicians into the national health care insurance plan in Japan, a smoking cessation program was initiated at Suidobashi Hospital, Tokyo Dental College, in 2006. The program is a new multi-disciplinary approach to smoking cessation, which includes interventions by the medical and dental professionals. While the program has been a significant addition to our patient care [17], it was our general feeling that more effort was needed to implement smoking cessation care to patients receiving dental treatments. As part of an effort to further promote smoking cessation activities, this study assessed smoking-related perceptions, attitudes and practices of dental school hospital-based health professionals.

## Methods

A cross-sectional survey of health professionals was conducted at Suidobashi Hospital, Tokyo Dental College, Tokyo, Japan in July 2010. Among the practicing professionals, those who were providing direct care to patients were asked to participate in the study.

### Questionnaire

A novel questionnaire was developed in order to assess smoking-related perceptions, attitudes and practices of health professionals. Specifically, the basic structure of the questionnaire items was generated in reference to the published studies [13,18] and to the Clinical Practice Guideline by U.S. Department of Health and Human Services [19]. Where required, one dentist, fluent in both Japanese and English performed the forward translation. A survey study in Japan [18] was also used as a guide for translation, as some of the wording in the questionnaire was similar to the material being translated. The base questionnaire was piloted with a small group of health professionals (two dentists, one dental hygienist, one nurse and one physician) in order to ensure the clarity and comprehensiveness of the questionnaire. The final and refined version (See additional file 1: Items in the survey questionnaire) was composed of the following 11 items. In Items 1 to 4, participant demographics were assessed. In Item 5, smoking status and history were requested. Items 6 and 7 inquired about participants' experiences with smoking cessation care training and their willingness to receive it. In Item 8, the provision of smoking assessment or cessation care to patients was assessed, with four subscale items on a 10-point scale, asking the participants how often they performed the intervention specified from never (0%) to always (100%). Where appropriate, the answers were dichotomised; the cut-off point was a median score of 50%. In Item 9, we assessed participants' perceptions regarding smoking or smoking cessation care, with seven subscale items on a 5-point scale, asking if they strongly disagree (1) or agree (5) with a given statement. Where appropriate, the answers were dichotomized ('strongly disagree', 'somewhat disagree', 'not sure either way' *versus *'somewhat agree' and 'strongly agree'). In Item 10, the participants' potential barriers were assessed. In the final Item 11, the participants were asked to express their views on smoking or smoking cessation care.

### Ethical considerations

Approval for the study protocol and the survey implementation was obtained from the Suidobashi Hospital's review board. A formal ethics review was waived by the institutional review board, because the research involved submitting a questionnaire to adults who complied of their own free will and, any information that would identify participants was avoided. Written informed consent was obtained from all participants.

### Statistical analysis

All the data obtained was examined and the responses were coded. The data was then descriptively analyzed and an appropriate test was applied, using a statistical package, InStat 3.1 (GraphPad, La Jolla, CA). Chi-squared test and one-way analysis of variance (ANOVA) with Tukey-Kramer multiple comparisons test were used.

A p value of less than 0.05 was considered as statistically significant.

## Results

### Characteristics of study participants

In total, 129 questionnaires were distributed, of which 105 were returned. Of these 93 (72%) were fully completed responses from practicing professionals.

Demographic data were listed in Table 1. The participants comprised predominantly oral health professionals; 58% were dentists and 24% were dental hygienists. 16% were registered nurses. Only small number of physicians (n = 2) participated, mainly because of the dental school-based hospital setting. Therefore, data for nurses and physicians were pooled to represent medical professionals for the subsequent analysis.

**Table 1:** Demographic characteristics and smoking status of the sample

	Overall (n = 93)	Dentist (n = 54)	Dental hygienist (n = 22)	Medical (n = 17)
Gender				
Male	35 (37.6)	35 (64.8)	0 (0)	0 (0)
Female	58 (62.4)	19 (35.2)	22 (100)	17(100)
Age (in years)				
20 - 29	30 (32.3)	20 (37.0)	10 (45.5)	0 (0)
30 - 39	33 (35.5)	20 (37.0)	4 (18.2)	9 (52.9)
40 - 49	15 (16.1)	5 (9.3)	6 (27.3)	4 (23.5)
50 - 59	12 (12.9)	8 (14.8)	1 (4.5)	3 (17.6)
60 ≤	3 (3.3)	1 (1.9)	1 (4.5)	1 (5.9)
Professional experience (in years)				
< 2	16 (17.0)	12 (22.2)	4 (18.2)	0 (0)
2 - 4	15 (16.1)	12 (22.2)	3 (13.6)	0 (0)
5 - 9	21 (22.6)	14 (25.9)	4 (18.2)	3 (17.6)
10 - 19	16 (17.0)	4 (7.4)	3 (13.6)	9 (52.9)
20 ≤	25 (26.9)	12 (22.2)	8 (36.4)	5 (29.4)
Smoking				
Current	17 (18.3)	15 (27.7)	2 (9.1)	0 (0)
Previous	14 (15.0)	10 (18.5)	4 (18.2)	0 (0)
Never	62 (66.7)	29 (53.7)	16 (72.7)	17 (100)

Data were shown as number (%)

Overall, nearly two-thirds (62%) of participants were women. About 65% of dentists were men, but all other professionals comprised women. About two-thirds of participants were aged 20-39 years. There was wide variety in the number of years after receiving professional qualifications. All medical professionals had at least 5 years of practicing experience, while 41% of dental professionals had fewer than 4 years of experience.

Altogether, 18% of participants reported being current smokers (17% of men and 19% of women), 15% reported being ex-smokers and 67% reported never smoking. Higher smoking rates were reported by dentists compared with other professionals (p = 0.0199, Chi-squared test for independence). None of the physicians or nurses who participated in the study was current smoker.

### Past training in smoking cessation care

The majority of participants reported that they have had never received training in smoking cessation care. Only 8% had previous training, and 71% indicated that they would like to receive it.

### Smoking assessment or cessation care

We next assessed the provision of smoking assessment or cessation care to patients. Among the four components of a brief intervention, 'asking smoking status' was the most frequently performed activity, followed by 'advise to quit'. (Table 2). Dentists and medical professionals asked about smoking status significantly more than dental hygienists (p < 0.05, p < 0.01, respectively; one-way ANOVA with Tukey-Kramer multiple comparisons test). Medical professionals were more likely to advise their patients to quit smoking than dental hygienists (p < 0.05).

**Table 2:** Provision of smoking assessment or cessation care

	Overall (n = 93)	Dentist (n = 54)	Dental hygienist (n = 22)	Medical (n = 17)
Asking smoking status	66.9 (36.2)	68.5 (36.3)*	46.4 (32.3)	88.2 (27.2) **
Advise to quit	43.9 (36.5)	43.3 (36.4)	30.9 (25.6)	62.4 (42.8)*
Assess willingness to make a quit attempt	22.5 (28.3)	23.3 (28.9)	22.2 (23.1)	20.0 (33.5)
Assist in quit attempt	17.3 (25.2)	14.6 (21.0)	19.1 (21.6)	23.5 (39.0)

Data were shown as mean % (SD)

*p < 0.05, **p < 0.001, significantly different from dental hygienist; one-way analysis of variance (ANOVA) with Tukey-Kramer multiple comparisons test

'Assess willingness to make a quit attempt' and 'assist in quit attempt' were the interventions implemented for less than one-quarter of their patients. There were no statistically significant differences in any of those interventions among the health professionals. There were no significant differences in provision of smoking assessment or cessation care between smokers and non-smokers, as assessed by Chi-squared test.

### Perception of smoking and smoking cessation

The participants' perceptions regarding smoking or smoking cessation care were assessed (Table 3). There was a statistically significant difference among overall scores (p < 0.0001). 'It is important to ask smoking status' and 'oral health professionals should participate more in smoking cessation care' were the subscale items with relatively high scores.

**Table 3:** Perception of smoking and smoking cessation

	Overall (n = 93)	Dentist (n = 54)	Dental hygienist (n = 22)	Medical (n = 17)
Q9-1 We should set an example by not smoking	3.9 (1.2)*	3.7 (1.3)	4.2 (0.7)	4.0 (1.0)
Q9-2 It is important to ask smoking status	4.6 (0.7)*	4.6 (0.8)	4.5 (0.5)	4.7 (0.5)
Q9-3 Most patients would not quit smoking anyway	3.1 (0.9)*	3.2 (0.8)	3.1 (0.9)	2.8 (1.1)
Q9-4 It is not easy to quit smoking because many smokers are addicted to nicotine	4.1 (0.9)*	4.1 (0.9)	4.2 (0.8)	4.0 (1.1)
Q9-5 Oral health professionals should participate more in smoking cessation care	4.1 (0.9)*	4.0 (0.9)	4.0 (0.7)	4.4 (0.7)
Q9-6 Oral health professionals' time would be better spent on other activities	2.6 (1.0)*	2.8 (1.0)	2.4 (0.8)	2.5 (1.2)
Q9-7 Dental patients have other important needs, so they have no time for smoking cessation care	2.3 (0.9)*	2.4 (0.9)	2.2 (0.7)	2.1 (1.0)

Data are shown as mean score (SD) from the rating scale of 1-5. A higher score indicates greater agreement with the statement.

*Significantly different between subscale items, p < 0.0001, ANOVA with Tukey-Kramer multiple comparisons test

There were no significant differences in these perceptions between professional types. When the relationship with smoking status was assessed, a statistically significant difference was noted in responses to 'oral health professionals should participate more in smoking cessation care' (p = 0.0404, Chi-squared test). Non-smokers were more likely to agree with this notion than smokers.

### Perceived barriers to smoking cessation care

**Figure 1:**
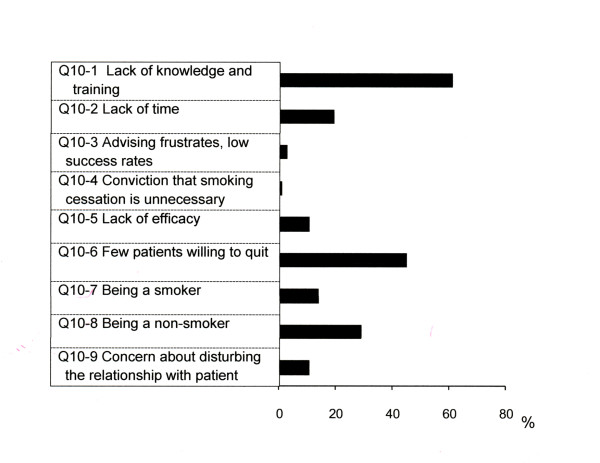
**Perceived barriers to smoking cessation care**. Data were shown as % response (the sum of percentages exceeds 100% because multiple answers were allowed)

'Lack of knowledge and training' (61%) was identified as a central barrier to smoking cessation care, followed by 'few patients willing to quit' (45%) (Figure 1). No statistically significant associations were noted between the demographic aspects including years in practice and the barriers that limit the health care professionals from providing smoking cessation care.

While many of the individual comments in Item 11 were related to the perceived barriers expressed in the previous item, they also reflected a general positive attitude toward provision of smoking cessation care.

## Discussion

This study aimed to identify smoking-related perceptions and practices of health care professionals at a dental-school based hospital in Japan. Despite a general positive perception towards smoking cessation, the provision as well as the extent of smoking cessation care by the study participants was found to be relatively low. The findings of the present study support the notion that potential of oral health professionals in smoking cessation is underutilized [7-14].

Oral health professionals have a role in encouraging their patients to quit smoking and helping them, via referral pathways. Also, dental staff should be trained and given the resources to deliver the advice themselves [20]. By the mid 1990's, a quarter of tobacco users in the United States had been advised to quit by their dentist [21]. However, even in northern Europe and North America, a substantial proportion of dental professionals either do not provide smoking cessation interventions or provide them on a very limited basis [22,23]. Little information is available on smoking cessation practices of oral health professionals in Asia. In a survey study of 152 dentists practicing in Malaysia [24], only 17.9% of them were actually involved in smoking cessation counselling. Consistent with these reports, the dentists and dental hygienists in the present study indicated that smoking cessation interventions such as 'assess willingness to make a quit attempt' and 'assist in quit attempt' were implemented for less than one-quarter of their patients who smoke. The efficacies of smoking-cessation intervention in dental or medical-dental settings were shown in Japan [4,17], suggesting the feasibility of integrating dentists and dental hygienists in a medical smoking cessation intervention. Encouragingly, in a recent survey study of 435 certified periodontists of the Japanese Society of Periodontology, a relatively high percentage (54.7%) of the participants indicated that they provide some form of smoking cessation care to their patients [25]. Initiatives are needed to further promote smoking cessation care in our practice setting.

Smoking behaviour among health professionals can be a significant obstacle in promoting smoking cessation initiatives [13,22]. A survey conducted among members of the Japan Medical Association in 2008 showed that the prevalence of cigarette smoking among 3,486 Japanese physicians was 15.0% for men and 4.6% for women [26]. A recent survey study conducted among 1000 dentists in Japan revealed that the prevalence of regular smokers was 25% for men and 3% for women [27]. In the present study, prevalence of smokers among women (19%) was relatively higher than that reported for the general population [5] or for Japanese dentists in private practice [27]. However, this needs to be interpreted with caution, since nearly two-thirds of the participants were women. The overall prevalence of smokers was higher than that (14.7%) reported for the certified periodontists [25]. Tobacco-using health professionals, including dentists, were reported to be less proactive than their non-using counterparts [15,28-30]. This trend was supported by our finding that non-smokers were more likely to feel that oral health professionals should participate more in smoking cessation care, when compared to smokers. These results suggested that further effort in smoking cessation among the health professionals in our hospital is needed.

We found that interventions by the dental hygienists in smoking cessation care were somewhat limited when compared to other health professionals (Table 2). This may be due to the fact that the dental hygienists working in our hospital have not been formally integrated into the smoking cessation treatment program. Given the importance of the role of oral health personnel in smoking prevention and control, an active participation of dental hygienists in the smoking cessation treatment program is necessary.

In the present study, lack of training and knowledge was considered to be the major barrier that potentially limited the involvement of the health professionals in smoking cessation activities. Also, more than one third of the participants perceived that few patients would be willing to quit. Removing such barriers can be difficult [31]. A Cochrane Review [32] on the training of health professionals in smoking cessation concluded that health professionals who were trained were better at delivering smoking cessation interventions. In the present study, no statistically significant associations were noted between the barriers and the demographic aspects including years in practice. Given that only 8% of the participants had prior training in smoking cessation care, it is necessary to offer opportunities for adequate training.

Rosseel et al. [13] reported that an atmosphere supportive of advice among colleagues is important in the implementation of strategies which support smoking cessation. Support from our own practice members and organization may increase the efficacy of the health professionals in smoking cessation care.

This study has several inherent limitations. The data were collected via a self-reported questionnaire. Like all questionnaires, the possibility of both intentional and unintentional mis-reporting threatens the validity and reliability of the findings. Relatively small number of participants allowed limited statistical analysis of data.

However, the present study adds to the existing literature by providing the salient information regarding the perceptions of and potential barriers to smoking cessation care among multiple health professions in Japan.

## Conclusions

We identified a need for further promotion of smoking cessation activities by the hospital-based health professionals. Hospital policies and faculty education providers need to provide staff with appropriate training and create an atmosphere supportive of smoking cessation activities.

## Consent

Written informed consent was obtained from the participants for publication of this manuscript and accompanying materials. A copy of the written consent is available for review by the Editor-in-Chief of this journal.

## Competing interests

The authors declare that they have no competing interests.

## Authors' contributions

AS designed the study, constructed the survey questionnaire, performed data analyses, and drafted the manuscript. AM, FU and MK contributed to data collection and analysis. MN interpreted the study and edited the manuscript. TM and TI oversaw procedures. All authors have read and approved the manuscript.

## Supplementary Material

Additional file 1 **The items in the survey questionnaire**. Click here for file
