# Supplementary material for: Health professional's perceptions of and potential barriers to smoking cessation care: a survey study at a dental school hospital in Japan
Source: BMC Res Notes. 2010 Dec 7;3:329. doi: 10.1186/1756-0500-3-329 (PMC3016266; doi:10.1186/1756-0500-3-329)
Supplement: Additional file 1 — The items in the survey questionnaire. [file 1756-0500-3-329-S1.DOC]

Items in the Survey Questionnaire

**Q1 Sex**

**Q2 Age**

**Q3 Profession**

**Q4 Professional experience**

**Q5 Smoking status**

**Q6 Past training in smoking cessation care**

**Q7 Willingness to receive training in smoking cessation care**

**Q8 Provision of smoking assessment or cessation care**

(1) Asking smoking status

(2) Advise to quit

(3) Assess willingness to make a quit attempt

(4) Assist in quit attempt

**Q9 Perception of smoking and smoking cessation**

(1) We should set an example by not smoking

(2) It is important to ask smoking status

(3) Most patients would not quit smoking anyway

(4) It is not easy to quit smoking because many smokers are addicted to nicotine

(5) Oral health professionals should participate more in smoking cessation care

(6) Oral health professionals’ time would be better spent on other activities

(7) Dental patients have other important needs, so they have no time for smoking cessation care

**Q10 Perceived barriers to smoking cessation care**

(1) Lack of knowledge and training

(2) Lack of time

(3) Advising frustrates, low success rates

(4) Conviction that smoking cessation is unnecessary

(5) Lack of efficacy

(6) Few patients willing to quit

(7) Being a smoker

(8) Being a non-smoker

(9) Concern about disturbing the relationship with patient

**Q11 Additional comments**
